# The Neural and Behavioral Correlates of Anomia Recovery following Personalized Observation, Execution, and Mental Imagery Therapy: A Proof of Concept

**DOI:** 10.1155/2018/5943759

**Published:** 2018-08-01

**Authors:** Edith Durand, Pierre Berroir, Ana Inés Ansaldo

**Affiliations:** Centre de Recherche de l'Institut Universitaire de Gériatrie de Montréal (CRIUGM), École d'Orthophonie, Faculté de Médecine, Université de Montréal, Montreal, QC, Canada

## Abstract

The impact of sensorimotor strategies on aphasia recovery has rarely been explored. This paper reports on the efficacy of personalized observation, execution, and mental imagery (POEM) therapy, a new approach designed to integrate sensorimotor and language-based strategies to treat verb anomia, a frequent aphasia sign. Two participants with verb anomia were followed up in a pre-/posttherapy fMRI study. POEM was administered in a massed stimulation schedule, with personalized stimuli, resulting in significant improvement in both participants, with both trained and untrained items. Given that the latter finding is rarely reported in the literature, the evidence suggests that POEM favors the implementation of a word retrieval strategy that can be integrated and generalized. Changes in fMRI patterns following POEM reflect a reduction in the number of recruited areas supporting naming and the recruitment of brain areas that belong to the language and mirror neuron systems. The data provide evidence on the efficacy of POEM for verb anomia, while pointing to the added value of combined language and sensorimotor strategies for recovery from verb anomia, contributing to the consolidation of a word retrieval strategy that can be better generalized to untrained words. Future studies with a larger sample of participants are required to further explore this avenue.

## 1. Introduction

Aphasia is an acquired language impairment following brain damage, such as stroke, whose consequences can be devastating [1]. Anomia is the most frequent and pervasive symptom for people with aphasia, regardless of the aphasia type. Anomia is described as difficulty in retrieving words in structured tasks, such as picture naming, sentence completion, or spontaneous speech. Anomia can affect different types of words, including nouns and verbs. Research has long focused on noun retrieval, while therapies targeting verb anomia remain rare [2]. This is somewhat surprising, considering the central role of verbs in sentence and speech production [3].

In recovery from aphasia, the attempt to compensate for anomia may be related to the concept of neuroplasticity. Neuroplasticity refers to a number of brain mechanisms involved in learning and relearning and is reflected in changes in brain activation patterns highlighted by functional magnetic resonance imaging (fMRI). Two main forms of neuroplasticity have been studied in the context of aphasia recovery: functional reactivation, which occurs when previously damaged and inactive areas recover their function after a latency period, and functional reorganization, which reflects compensation for the permanent damage of specific brain areas by the recruitment of other areas not previously involved in the given function [4]. Different types of neuroplasticity may be involved in recovery from anomia; adaptive neuroplasticity results in functional recovery, whereas maladaptive neuroplasticity results in persistence of errors [4, 5]. There is a long-standing debate in the anomia recovery literature regarding functional reorganization: Is better recovery supported by perilesional left hemisphere (LH) language processing areas or right hemisphere (RH) homologues of those areas? However, the extent to which an RH shift reflects adaptive or maladaptive neuroplasticity remains controversial (Anglade et al., 2014). Moreover, the impact that different therapy procedures may have on the recruitment of canonical or noncanonical language processing circuits remains to be explored.

With regard to verb anomia, therapy approaches have been designed with reference to models of word processing that view the phonological and semantic processing of words as key elements for word retrieval (see [2], for a review). Thus, phonological approaches use sound cues and rhymes to elicit words, whereas semantic approaches use semantic cues and reinforce the semantic features of a given word to facilitate word naming. The efficacy of both approaches has been proven, in particular with treated items [2]. Conversely, poor generalization of treatment effects to untrained verbs has been consistently reported [6–12]. Furthermore, none of these studies have explored the neural substrates sustaining recovery from verb anomia. Regarding the lack of generalization of therapy effects to untrained verbs, it should be noted that none of the publications cited took into consideration the dynamic component of verb processing. The meaning of an action verb includes a dynamic semantic feature that an object does not require. This assumption—grounded in embodied cognition theory—implies that word meaning depends on modal experiences. Thus, semantic processing of a given word—noun or verb—will depend upon the sensory and motor modalities by which objects and actions corresponding to those words are learned and how this learning impacts the functional brain networks supporting word processing ([13, 14]; Pulvermüller et al., 1996). In other words, the learning modality and features of a given word will determine the conceptual and brain-related substrates supporting word retrieval; with verbs, particularly action verbs, these should include sensorimotor features and brain processing areas [15].

An interesting example of how word encoding influences the efficacy of a given strategy for word retrieval comes from the work by Marangolo et al. showing that action observation on its own can represent a useful tool for verb retrieval [16, 17]. Action observation therapy (AOT) principles were first developed for stroke patients who suffered from a motor deficit affecting the upper limbs. Several studies have consistently shown that AOT is an effective way to enhance motor function [18–21]. Ertelt et al. [18] first showed that patients in the chronic stage after stroke experienced significantly improved motor function following a four-week video therapy program compared with a control therapy; additionally, neural activations associated with the AOT showed a significant rise in activity in areas sustaining the action observation/action execution matching system [18]. This system includes the mirror neuron system, which will be discussed below.

In the language rehabilitation domain, Marangolo et al. [17] administered AOT to stroke patients who suffered from aphasia in order to improve verb retrieval. They compared action observation with action observation and execution and found that the mere observation of the performed action was sufficient to activate the corresponding sensorimotor representation in the semantic system, which served as input at the lexical level facilitating verb retrieval. However, their results were not replicated by another recent work [22] and the effect was restricted to trained items. Moreover, the neural substrate underlying recovery with AOT has not yet been investigated.

Several studies have examined the efficacy of other sensorimotor strategies to facilitate verb retrieval. For example, Raymer et al. (2006) examined the effect of gesture execution in aphasia treatment, using pantomimes paired with verbal training for noun and verb retrieval in a group of aphasic patients. Their results showed improved naming of trained nouns and verbs but no generalization of treatment effects to untrained words. Similarly, Rose and Sussmilch [23] obtained significant results following therapy combining verb naming and gesture production; again, the results were restricted to trained items. In sum, observation of action and gesture execution, both associated with verb naming, yielded positive results with trained verbs but not with untrained ones. None of those studies included fMRI segregation analysis of areas sustaining recovery, and thus the behavioral changes observed cannot be linked to any specific neural substrate. Thus, while functional neuroimaging data on verb processing have mostly been related to healthy populations, very little is known about therapy-induced neuroplasticity in the recovery from verb anomia.

In healthy adults, action verb naming has been shown to be supported by left frontal cortical areas, including the left prefrontal cortex (Shapiro et al., 2001), the left superior parietal lobule, the left superior temporal gyrus (Shapiro et al., 2006), the left superior frontal gyrus (Shapiro et al., 2005), and the primary motor cortex in the posterior portion of the precentral gyrus (Porro et al., 1996, [13], and Pulvermüller et al., 2005). As discussed by Durand and Ansaldo [15], these areas have also been associated with the so-called mirror neuron system (MNS), which is thought to support AOT in motor neurorehabilitation after stroke. Mirror neurons are a particular class of visuomotor neurons, originally discovered in area F5 of the monkey premotor cortex, that discharge both when a monkey does a particular action and when it observes another monkey or a human doing a similar action [24]. The MNS is a mechanism that unifies perception and action, transforming sensory representations of the behavior of others into motor representations of the same behavior in the observer's brain [25]. From this perspective, some authors have suggested that language evolved from a gestural system, first as pantomime and gradually as conventional gestures, eventually developing into a symbolic code [24, 26, 27]. This sensorimotor system is considered to be the structure underlying vocabulary and grammar development [26, 28]. In this view, mirror neurons are considered to be embodied cognitive agents, as they coordinate multimodal information resulting from an individual's interaction with the environment. According to such theories, the MNS may play a central role in the development of language in humans [24, 26, 27] and in semantic processing, especially action semantic processing.

Apart from the MNS, several links can be made between vision and action. The cortical visual system is known to be segregated into two anatomically and functionally distinct pathways: a ventral occipitotemporal pathway that subserves object perception and a dorsal occipitoparietal pathway that subserves object localization and visually guided action [29–31]. Goodale and Goodale and Milner [30, 32] proposed a model in which the perceptual detection of possible actions in the environment involves the dorsal stream, stretching from the primary visual cortex to the posterior parietal lobe and reaching the premotor areas and a distributed network of areas in the caudal frontal cortex. More than just a visual detection system, the dorsal stream allows action selection with continuous matching between the visual and motor areas [33]. A recent study has shown that, along the dorsal pathway, the anterior intraparietal area and the ventral premotor cortex extract sensorimotor information from perceptual stimuli, making it possible to detect action possibilities from the information detected through the retinotopic map [33].

Recent research shows that sensorimotor processes play a crucial role in language processing. Thus, both behavioral studies [34] and neurofunctional studies [35–44] suggest that the understanding of action words recruits motor areas. Along the same lines, Tremblay and Small [44] showed that functional specialization of specific premotor areas is involved in both action observation and execution. Moreover, Tomasino and Rumiati (2013) showed that the involvement of sensorimotor areas depends on the strategy used to perform the task. Specifically, if the task requires a person to imagine actions, sensorimotor areas will be involved. Visual mental imagery allows one to obtain an internal representation that functions as a weak form of perception [45]. Mental imagery is known to be an efficient therapy tool for rehabilitation of motor impairments. In language rehabilitation, mental imagery is a relatively new tool, though some studies on aphasia recovery report the activation of visual mental imagery processing areas, such as the inferior occipital gyrus [46].

Taking into account the promising but limited results obtained with anomia therapy approaches based on action observation, gesture, or mental imagery used separately, we designed a new therapy approach combining three sensorimotor strategies previously used to treat verb anomia, namely, action observation, gesture execution, and mental imagery, and combined the three of them in a massed practice format. Thus, personalized observation, execution, and mental imagery therapy (POEM therapy) was designed based on principles of experience-dependent neuroplasticity, namely, stimulus specificity and salience, and a time/frequency ratio corresponding to massed stimulation (for a review of this issue, see [5]). Several studies have shown the benefits of massed practice, defined as practice of a given number of trials in a short time [47–49].

In sum, POEM therapy was developed based on evidence, while incorporating principles of experience-dependent neuroplasticity and targeted, repetitive, and intensive practice of action naming, with the purpose of contributing to strategy development and integration [5]. Moreover, to identify the neural substrates associated with the outcomes of POEM therapy, we used fMRI to assess functional brain activity before and after intervention with POEM therapy and thus assess treatment-induced neuroplasticity.

The purpose of this study is to examine the effects of POEM therapy on the recovery from verb anomia in the context of chronic aphasia and to identify the neural changes associated with behavioral improvement. Two participants with chronic nonfluent aphasia were examined before and after POEM therapy, and behavioral and event-related fMRI measures were taken. Participants received three sessions of POEM therapy per week over five weeks, in line with a massed therapy approach [47, 48, 50]. Activation maps obtained in the context of oral verb naming were obtained before and after POEM therapy. It was expected that

1. POEM therapy would result in significant recovery of verb naming;
2. a series of motor and premotor areas would sustain the observed recovery.

## 2. Material and Methods

### 2.1. Participants

**Figure 1:**
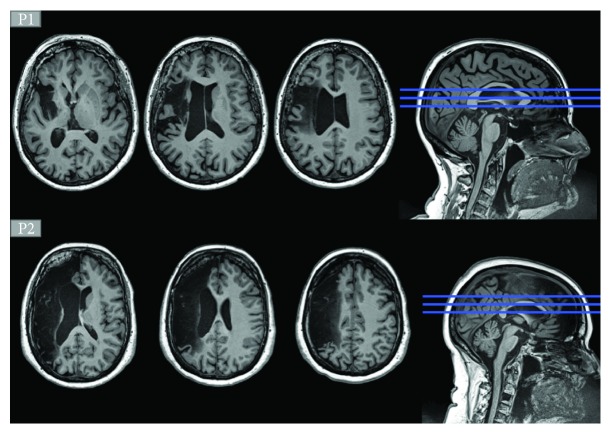
Lesion location on anatomical MRI for P1 (top three slices) and for P2 (bottom three slices).

Aphasia severity and typology were determined by an experienced speech-language pathologist (SLP: ED). Inclusion criteria were (1) a single LH stroke, (2) a diagnosis of moderate-to-severe aphasia according to the Montreal-Toulouse Battery (Nespoulous et al., 1986), (3) the presence of anomia according to a standardized naming task [51], (4) having French as their mother tongue, and (5) being right-handed prior to the stroke (Edinburgh Inventory; Oldfield, 1971). Exclusion criteria were (1) the presence of a neurological or psychiatric diagnosis other than stroke, (2) incompatibility with fMRI testing, or (3) diagnosis of mild cognitive impairment or dementia prior to stroke [52]. Participants gave written informed consent according to the Declaration of Helsinki. This study was approved by the Ethics Committee of the Regroupement de Neuroimagerie Québec.  Table 1 contains sociodemographic information on the two participants, and Figure 1 shows their structural magnetic resonance imaging (MRI) results.

#### 2.1.1. Participant 1

P1 is a 65-year-old right-handed woman, who was 7 years postonset from a left temporal stroke, which resulted in nonfluent aphasia and right hemiparesia. She benefited from individual language therapy for a short time just after the stroke; since then, she has participated in activities organized by the association for persons with aphasia. At the beginning of the study, she was not receiving any language therapy. Aphasia testing conducted at that point showed moderate transcortical motor aphasia with moderate apraxia of speech.

#### 2.1.2. Participant 2

P2 is a 72-year-old right-handed woman, who was 34 years postonset from a left temporal stroke, which resulted in nonfluent aphasia and right upper limb hemiplegia. She had received individual language therapy intermittently over the previous 20 years, particularly during the first years after the stroke. She often participates in activities organized by the association for persons with aphasia. At the beginning of the study, she was not receiving any language therapy. Aphasia testing conducted at that point showed severe transcortical motor aphasia with mild apraxia of speech.

### 2.2. Experimental Procedure

The experimental protocol is similar to previous studies conducted in our lab (Marcotte and Ansaldo, 2010, 2012, and 2013). A baseline language assessment was conducted prior to therapy, followed by an initial fMRI session (T1), which identified the neural substrate of spontaneous correct naming. Afterward, patients received therapy from a trained SLP (ED). A second fMRI session (T2) was performed after five weeks of therapy. This session allowed us to identify the brain areas that subserved therapy-induced neuroplasticity. During both fMRI sessions, patients performed an overt naming task. (See Table 2 for the MRI results.)

#### 2.2.1. Language Assessment

Before therapy, the participants were examined with subtests from Montreal-Toulouse 86 Beta version (Nespoulous et al., 1986) to assess global comprehension, repetition, and fluency; the kissing and dancing test (KDT) for verb comprehension [53]; the dénomination de verbes lexicaux (DVL38) for verb naming [51]; the test de dénomination de Québec (TDQ) for noun naming [54]; and three subtests of the Apraxia Battery for Adults—Second Edition [55]—to measure the presence and severity of verbal, limb, and oral apraxia. These tests allow a complete description of the aphasia profile.

#### 2.2.2. Baseline and Items for fMRI Session and Therapy

Stimuli used for the baseline, the fMRI naming task, and the therapy sessions were 5-second action videos (Durand et al., in prep.). Before therapy, the participant underwent three baseline naming assessments using 134 action videos. Baselines were separated by at least four days; the participant had to show stable oral naming performance. In order to provide more individualized therapy, a set of stimuli was created for the participants on the basis of individual performance on the baseline as follows: correctly named (spontaneous, *n* = 20) and incorrectly named (*n* = 60). Of the incorrectly named items, only 20 were trained and the remaining 40 items allowed us to measure the generalization of therapy effects to untrained items. All sets of items (spontaneous, trained, and untrained) were matched for word frequency, number of phonemes, and syllabic complexity. Statistical analysis of the lists showed nonsignificant differences regarding these variables.

Before the first fMRI session, each participant took part in a practice session in a mock scanner. They could therefore become accustomed to the scanner noise and environment.

For the pretherapy fMRI sessions, a set of items was developed including correctly named (spontaneous, *n* = 20) and incorrectly named (*n* = 60) items and scrambled videos that were optimized to fit the same parameters (motion, colors) as the videos for the control conditions (*n* = 40). For the posttherapy fMRI session, the same set was presented, but this time, the incorrectly named items (*n* = 60) were divided into trained items (*n* = 20) and untrained items (*n* = 40) to measure generalization.

During the fMRI scanning, participants were instructed to name the randomly presented videos and to say “baba” in response to scrambled videos. After therapy, the same set of items was presented. Oral responses were audio-recorded with Audacity software.

#### 2.2.3. fMRI Sessions

**Figure 2:**
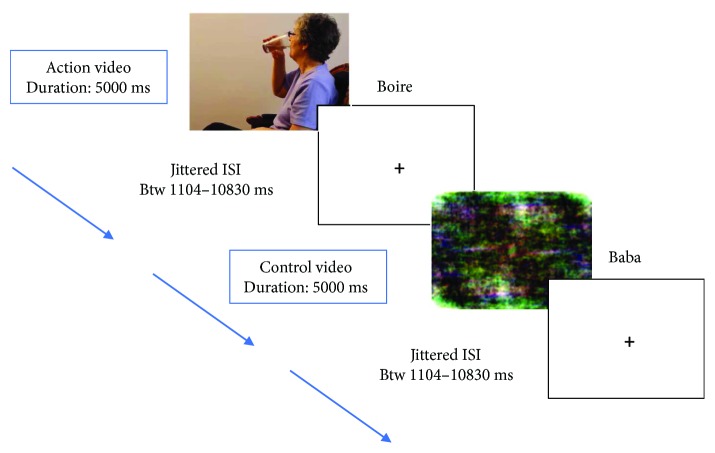
Naming task during fMRI acquisition.

Participants lay in a supine position on the MRI scanner bed with their head stabilized by foam. Stimuli were pseudorandomly displayed in an optimized order projected by means of E-Prime software (Psychology Software Tools) from a computer onto a screen at the head of the bore and were visible in a mirror attached to the head coil. Each video and picture was presented for 5000 ms, with an interstimulus interval (ISI) ranging from 1104 to 10,830 ms. As shown in Figure 2, participants were instructed to name each action and object, as clearly and accurately as possible, and to say “baba” each time they saw a distorted picture, while avoiding head movements. An MRI-compatible microphone was placed close to the participant's mouth, and Audacity software (http://www.audacityteam.org) was used to record oral responses.

#### 2.2.4. Functional Neuroimaging Parameters

Images were acquired using a 3T MRI Siemens Trio scanner, which was updated (Prisma Fit) during our data collection, with a standard 32-channel head coil. The image sequence was a T2^∗^-weighted pulse sequence (TR = 2200 ms; TE = 30 ms; matrix = 64 × 64 voxels; FOV = 210 mm; flip angle = 90°; slice thickness = 3 mm; and acquisition = 36 slides in the axial plane with a distance factor of 25% in order to scan the whole brain, including the cerebellum). A high-resolution structural image was obtained before the two functional runs using a 3D T1-weighted imaging sequence using an MP-RAGE (TFE) sequence (TR = 2300 ms; TE = 2.98 ms; 192 slices; matrix = 256 × 256 mm; voxel size = 1 × 1 × 1 mm; and FOV = 256 mm).

#### 2.2.5. Language Therapy with POEM

A trained SLP (ED) provided the POEM therapy, which lasted for one hour and was provided three times per week, over five weeks. During each session, participants were trained to name 20 actions presented in 5-second videos. If the participant could not name the action within 5 to 10 s, she was asked to make the gesture associated with this action, helped by the SLP. If she could not name the action, the participant was asked to imagine the action in a personal context. For instance, with the action *to water*, the following sequence can be produced after the action observation: the SLP says “Show me what the person is doing with your hands,” and the participant can imitate someone who is watering. If the action is still not named, the SLP says “Imagine this action in your garden.” After these prompts, the word was given to the participant, who was asked to repeat it once.

### 2.3. Behavioral and fMRI Data Analysis

Responses to the fMRI naming task were recorded and coded offline by an experienced SLP (ED), in order to build the design matrices. Preprocessing and statistical analyses were performed using SPM12 software (Wellcome Trust Centre for Neuroimaging, Institute of Neurology, University College London), running on MATLAB_R2016b (MathWorks Inc., MA, USA). fMRI images were preprocessed with the usual spatial realignment and slice timing. Motion was assessed to ensure that the naming task did not involve head motion exceeding 3 mm. Because precise, valid normalization is critical to understanding the neural substrates of treatment-induced recovery, we used the “Clinical toolbox” extension [56]. This toolbox allows optimal segmentation and registration of brains with distorted anatomy due to lesions. Lesion masks (PB) hand-traced on T1-weighted images were used to minimize the impact of the lesion on the normalization estimates, by substituting healthy tissue for homologous regions of the intact hemisphere [57]. This yields transformation matrices for normalization into the standard stereotaxic space (MNI space) with 3 × 3 × 3 mm^3^ voxel size. A spatially smoothed 8 mm Gaussian filter was chosen for the smoothing step. Preprocessed data were analyzed using the general linear model implemented in SPM12. Statistical parametric maps were obtained for each subject and each measurement period (first and second fMRI sessions), by applying linear contrasts to the parameter estimates for the conditions of interest (successful naming with trained/untrained items). Neuroimaging data analyses were performed only on correct responses. Individual maps were calculated for each condition for the whole brain with cluster size superior to 10 voxels and *p* < 0.001 uncorrected.

Furthermore, a Lehéricy index (LI) was calculated for each participant to estimate the relative contribution of the LH and RH to verb naming in each condition, pre- and posttherapy. We applied Lehéricy's algorithm, defined as follows: (*L* – *R*)/(*L* + *R*), where *L* represents the number of activated voxels in the LH and *R* represents the number of activated voxels in the RH. LIs were calculated using voxels in clusters (*k* ≥ 10) that exceeded the threshold (*p* < 0.001 uncorrected). LIs can range from −1.0 to +1.0. By convention, values between −0.2 and +0.2 represent bilateral language distribution, values between −0.2 and –1.0 represent RH dominance, and values between +0.2 and +1.0 represent LH dominance. Values between ±0.5 and ±1.0 are considered to reflect strong hemisphere dominance [58].

## 3. Results

### 3.1. Participant 1

By the end of the therapy period, P1 was able to name all of the 20 trained items. However, her performance in the scanner was less accurate than that at the last therapy session, as she named 16 trained items in the posttherapy fMRI session, which occurred one day after the end of therapy. In addition, P1 named 30 of the 40 untrained items that she was unable to name before therapy. Moreover, P1 showed improved verb naming on the DVL38 and noun naming on the TDQ.

As for her fMRI results, spontaneous correct naming before therapy significantly activated the left primary motor cortex, left angular gyrus, and right fusiform gyrus, with predominant LH activation according to the LI. (See Table 3 for fMRI results and Table 4 for LIs.)

Regarding trained items after the therapy, the activation map revealed significant activation in the left cerebellum, left and right middle temporal gyri, and right fusiform gyrus. Moreover, the LI indicated an increase in predominant LH activation (0.17).

Finally, with untrained items, the posttherapy activation map showed significant activation of regions similar to those activated for the trained items, namely, the left middle temporal gyrus and right fusiform gyrus, with the addition of the right inferior frontal gyrus. The LI in this case showed a shift to predominant RH recruitment.

### 3.2. Participant 2

Following therapy, P2 was able to name all of the 20 trained items and correctly named 19 trained items in the posttherapy fMRI session. P2 also named 13 of the 40 untrained items she had been unable to name before therapy. Again, her performance outside the scanner was better for untrained items. Finally, like P1, P2 showed improved verb naming ability on the DVL38 and noun naming ability on the TDQ.

The activation map for correct naming before therapy showed the recruitment of a large set of areas, including bilateral activation of the angular gyrus, superior parietal lobule, premotor cortex, left middle and inferior occipital gyri, and right cerebellum. The LI (0.6) corresponded to a predominant LH activation. (See Table 3 for fMRI results and Table 4 for LIs.)

With trained items, posttherapy activation maps were much smaller, as fewer areas were recruited, namely, the right premotor cortex and left cerebellum, and the LI showed predominant RH activation (−0.58). Unfortunately, it was not possible to obtain an activation map for untrained items, due to the lack of a suprathreshold cluster number.

## 4. Discussion

This study examined the behavioral and neural correlates of personalized observation, execution, and mental imagery (POEM) therapy, a new approach combining sensorimotor and language-based strategies to treat verb anomia, which was delivered in a massed stimulation format. Two participants with nonfluent chronic aphasia were examined with a verb naming task during event-related fMRI scanning, before and after therapy. Both participants benefited from POEM, with improvements observed with both trained and untrained items. Concurrently with the behavioral improvement, changes in the neural substrates sustaining verb naming were observed in both participants, with distinctive activation patterns observed posttherapy, including areas related to the nature of POEM therapy.

As hypothesized, the outcomes revealed the positive effects of POEM therapy on verb naming for both participants. The results are in line with previous studies showing that sensorimotor strategies are efficient therapy tools for recovery from verb anomia secondary to aphasia [16, 17, 22, 23]. However, none of those studies found positive therapy effects on untrained items. Two possible interpretations of these results were considered: on the one hand, they could be due to the origins of verb anomia; on the other hand, they could be due to the types of strategies used. In their study using semantic plus gesture treatments for verb anomia, Rose and Sussmilch [23] reported significant improvement for two participants with lexical-phonological-based anomia, but there is no improvement for the participant with semantic-based anomia. Similarly, Marangolo et al. [17] obtained positive results with AOT on verb retrieval for participants with lexical-phonological-based verb anomia, but there is no improvement for those who presented semantic-based verb anomia. The authors of those studies suggested that the severity of the semantic impairment underlying the anomia was responsible for the lack of improvement after the therapy. In our study, the semantic processing assessment showed that each participant had a preserved semantic system before the therapy. Because sensorimotor strategies are related to the semantic component of action, the improvement in verb retrieval would have been facilitated by preserved semantic abilities.

Furthermore, improvement was also observed on the untrained list after POEM therapy. Although this result was limited for P2 in the context of fMRI, the improvement was noted behaviorally and the same result has been found consistently with a group of 10 participants who have received POEM therapy (Durand et al., in prep). However, a generalization to untrained items was not found in several earlier studies using sensorimotor strategies. The sensorimotor strategies applied by Marangolo et al. [17], Raymer et al. (2007), and Rose and Sussmilch [23] used only one type of sensorimotor cue—gesture or observation in association with verb naming—whereas with POEM therapy, several sensorimotor cues were provided—observation of the action, gesture, and mental imagery—which may have facilitated word retrieval. According to cognitive models of word naming, this combination of semantic inputs could increase activation at the semantic level and facilitate the flow to the lexical and articulation levels and verb naming [59, 60]. Moreover, in line with the embodied theory, the various sensorimotor cues in POEM therapy tap into the specific encoding features of verbs [14, 26, 36, 42, 44], thus enhancing the therapy's specificity, another factor that has been shown to contribute to therapy efficacy [2].

The personalized approach potentially contributes to POEM's efficacy and generalization effects. Thus, verbs targeted with POEM were selected according to each participant's naming performance before therapy. Personalization of therapy items is considered to increase motivation, and thus attention focus, and has been shown to contribute to therapy efficacy [61].

Finally, as shown by previous works [47–49], massed stimulation with the POEM protocol may also explain the differences observed between our study and the other studies considered. The structured and massed practice on a limited number of items may have contributed to the implementation of a naming strategy that could be generalized to untreated items.

The improvement observed for our two participants occurred concomitantly with changes in neural recruitment. As hypothesized, the recovery following POEM therapy involves the recruitment of an alternative circuit, including the activation of motor and premotor areas. Although the behavioral improvement looks the same for both participants, two different patterns appeared after the POEM therapy.

In the case of P1, the pretherapy fMRI session showed bilateral distribution according to the LI. More specifically, considering the activation maps for spontaneously named items to be trained or untrained, the recruitment includes the left primary motor area, left angular gyrus, and right fusiform gyrus. The left primary motor area and left angular gyrus are canonical areas, part of the dorsal stream pathway of language [62], that reveal the perilesional recruitment associated with aphasia recovery. These two areas are also known to be involved in verb naming [13, 63]. The angular gyrus, which is an associative area between somatosensory information and visual information, participates in the processing of sequence actions, which may be related to the processing of the action videos (Crozier et al., 1999). The recruitment of the right fusiform gyrus can also be related to the processing of visual stimuli. The fusiform gyrus is involved in lexical-semantic association, that is, associating words with visual stimuli [64]. To summarize, for P1, the pretherapy fMRI session revealed the recruitment of canonical areas for verb naming, including perilesional areas, in line with a functional reactivation.

After the POEM therapy, the activation map for trained items reveals that distribution is still bilateral (LI = 0.17), including the right fusiform gyrus and the bilateral middle temporal gyri and left cerebellum. The bilateral middle temporal gyri participate in semantic processing, word generation, and observation of motion [65]. Classically, the cerebellum is known to regulate motor movement and be involved in motor speech planning. But recent fMRI studies have revealed the contribution of the cerebellum to other kinds of language processing [66, 67], namely, verb generation [68]. To sum up, post-POEM therapy, the activation pattern is consistent with the sensorimotor nature of POEM therapy and therefore is likely to have been therapy-induced.

More interestingly, in P1, the activation patterns for trained and untrained items posttherapy included common areas, with the activation of the left middle temporal gyrus, right fusiform gyrus, and right inferior frontal gyrus. The similarity of neural recruitment for trained and untrained items after POEM therapy suggests that the same kind of processing was used to name the verbs. Furthermore, these similar activations occur concomitantly with the generalization observed in behavioral results. The behavioral and neural results are evidence of the potential application of the same strategies to retrieve verbs.

In the case of P2, the pretherapy fMRI session showed dominant LH activation according to the LI. Considering the large lesion on the left hemisphere, it is not surprising that the activation for spontaneously named items included posterior visual processing areas such as the striate cortex and middle and inferior occipital gyri. But canonical areas for verb naming were also recruited, namely, the angular gyrus and premotor cortex bilaterally. These areas are known to be part of the action naming network in the LH [13, 14, 44]. The bilateral activations on the activation map pretherapy revealed adaptive neuroplasticity with a functional reorganization, which included the homologous areas for verb naming.

After P2's POEM therapy, there was a dramatic decrease in the number of areas recruited for verb naming. The posttherapy activation is supported exclusively by the right premotor area and the left cerebellum. As discussed above, these two areas are involved in action observation and verb naming [44, 66, 67]. This significant reduction in the number of brain areas supporting correct naming suggests that POEM therapy could lead to a more economical use of brain resources. Moreover, considering the LI (−0.58), there was a shift to the RH. This shift is related to adaptive neuroplasticity and is not surprising considering P2's large lesion. This result is in line with the suggested complementary role of the RH in the context of large lesions proposed by Anglade et al. (2013) who argued that, when there is a large lesion with near-complete destruction of the primary language processing areas, significant RH activation is involved.

Our preliminary results showed that neural changes appeared together with behavioral improvements in verb naming after POEM therapy was applied. Although neurorehabilitation studies in the physical domain had provided convincing evidence that action observation and motor imagery might enhance the efficacy of motor training and/or motor recovery by stimulating the activity of the sensorimotor system [69–72], no studies had explored this combination in the case of language rehabilitation. However, the link between action observation, motor imagery, and the sensorimotor system through the MNS system may apply to language too. As discussed by Durand and Ansaldo [15], the MNS is considered to have provided a natural platform for the development of language in humans. Several studies in the field of embodied cognition have provided evidence that the sensorimotor system can be considered an embodied cognitive agent, as it coordinates multimodal information resulting from an individual's interaction with the environment and constitutes a physiological substrate for empirical data linking language and motor processing [24, 26, 27].

Several fMRI studies have shown links between language and motor processing areas within the MNS. Specifically, language comprehension and production tasks engage somatotopic activations, that is, the recruitment of specific motor areas, depending on the body part involved in the action associated with the language target [35, 43]. These findings suggest that the MNS plays an important role in the reintegration of sensorimotor representations during the conceptual processing of actions evoked by linguistic stimuli. Thus, the cooccurrence of these activations weaves connections between motor and language processing areas. These connections represent an interesting framework devoted to the enhancement of skill recovery in language rehabilitation. They were exploited through the application of POEM therapy, leading to preliminary results with two participants.

This work concerns two case studies, and thus, it represents a proof of concept for further investigation of the effects of POEM. Thus, larger experimental samples are required to test for the external validity of these findings. This being said, the two single-case studies reported here concern two different cases, in terms of lesion size, location, and volume, thus providing evidence for the efficacy of POEM in more than one type of aphasia patients. Hence, while group study strength lies on statistical power, single-case studies are informative in terms of the variables that can influence recovery. In particular, group studies average activations, while single-case studies show different patterns of neurofunctional changes, in particular perilesional activations, which are known to better correlate with functional recovery [73]. The present study shows how similar behavioral improvement across the two participants is observed in the context of different lesion volumes and neurofunctional patterns.

Another potential caveat of the present study concerns sociodemographic differences between the two participants, in particular, time poststroke, lesion volume, and education level. Specifically, P2 was 408 months poststroke, while P1 was 84 months poststroke. Time elapsed after stroke has been shown to play an important role in treatment-related changes, but this concerns particularly the acute or subacute phase of recovery, as opposed to the chronic state, which is generally considered to go beyond 6–12 months after stroke [74, 75]. Consequently, we do not think that differences in neurofunctional patterns observed in P1 and P2 can be accounted for by time elapsed after stroke but reflect the influence of lesion size and volume, while these two factors do not seem to modulate POEM therapy efficacy, as documented by equivalent improvement across the two participants.

In all, the results of this study provide evidence for the efficacy of POEM and its neural correlates, in two cases of chronic verb anomia, resulting from lesions varying in size, location, and volume, and in participants with different educational backgrounds. Future studies will examine the effects of POEM on larger samples (Durand et al., in prep.) and gather both the anatomical and functional correlates of language and motor networks sustaining its efficacy. It will possibly increase our understanding of the mechanisms underlying the recovery from verb anomia, so that more efficient and synergistic rehabilitative interventions based on the links between motricity and language can be designed.

## Figures and Tables

**Table 1:** Sociodemographic, clinical, and cognitive data for the 2 participants.

Patient ID	P1	P2
Sociodemographic data		
Age (years)	65	72
Gender	F	F
Education (years)	18	11
Clinical data		
Handedness	R	R
Etiology	Ischemia	Ischemia
Months postonset	84	408
Aphasia type	Transcortical motor	Transcortical motor
Lesion volume (cm^3^)	38	132
Level of verb anomia	68%	55%
Cognitive data (CASP)		
Language (max. 6)	5	6
Visuoconstructive functions (max. 6)	6	5
Executive functions (max. 6)	6	6
Memory (max. 6)	6	6
Praxis (max. 6)	6	5
Orientation (max. 6)	4	6
Total CASP (max. 36)	33	34

CASP: Cognitive Assessment scale for Stroke Patients (Benaim et al., 2015).

**Table 2:** Language assessment and verb naming scores during the pre- and posttherapy MRI sessions for both participants.

Patient ID	P1		P2	
Language assessment	Pre	Post	Pre	Post
Comprehension (max. 47)	46	45	32	N/A
Repetition (max. 33)	30	30	N/A	N/A
Fluency	11	5	15	16
TDQ (max. 60)	40	47	52	57
KDT (max. 52)	51	49	48	N/A
DVL38 (max. 114)	77	81	63	65
Verb naming scores during fMRI session	Pre	Post	Pre	Post
Score for trained items (/20)	9	16	10	19
Score for untrained items (/40)	24	30	15	13

Pre: pre-POEM therapy; Post: post-POEM therapy.

**Table 3:** Significantly activated areas associated with the production of correct verbs for the two participants.

Patient ID	Condition	Pretherapy														Condition	Posttherapy													
		Left hemisphere SPM results							Right hemisphere SPM results								Left hemisphere SPM results							Right hemisphere SPM results						
		Region	BA	X	Y	Z	*T*-score	Cluster size	Region	BA	X	Y	Z	*T*-score	Cluster size		Region	BA	X	Y	Z	*T*-score	Cluster size	Region	BA	X	Y	Z	*T*-score	Cluster size
P1	Spontaneously named > baba	Primary motor	4	−39	−25	65	4.82	20	Fusiform	37	60	−46	5	4.79	28	Spontaneously named > baba								Middle temporal gyrus	21	60	−43	2	4.2	19
		Angular gyrus	39	−60	−49	35	3.74	13																						

P1	Incorrectly named > baba	Angular gyrus	39	−60	−43	26	3.49	10	Fusiform	37	60	−46	5	4.2	15	Trained > baba	Cerebellum		−24	−88	−28	4.6	31	Fusiform	37	60	−49	5	4.42	32
			39	−60	−52	32	3.37										Middle temporal gyrus	21	−60	−22	−4	4.02	14	Middle temporal gyrus	21	48	−40	5	3.46	
																	Middle temporal gyrus	21	−54	−31	−1	3.52								
																Untrained > baba	Middle temporal gyrus	21	−60	−25	−4	4.68	21	Fusiform	37	60	−46	5	5.04	82
																	Middle temporal gyrus	21	−54	−31	−1	3.81		Inferior frontal gyrus	44	39	11	17	4.32	54

P2	Spontaneously named > baba	Angular gyrus	39	−27	−67	32	5.2	1117	Superior parietal lobule	7	33	−55	53	4.35	117	Spontaneously named > baba	Premotor cortex	6	−15	−19	50	4.5	59	Premotor cortex	6	51	−4	35	5.56	114
		Superior parietal lobule	7	−27	−67	44	5.08		Angular gyrus	39	30	−67	26	3.64			Middle occipital gyrus	18	−24	−94	2	3.49	20	Cerebellum		15	−73	−31	3.56	12
		Superior parietal lobule	7	−21	−61	35	4.84		Angular gyrus	39	33	−64	35	3.47			Middle occipital gyrus	18	−12	−85	−10	3.47	14							
		Inferior occipital gyrus	19	−33	−73	−4	4.76	167	Cerebellum		−18	−76	−19	3.95	115		Cerebellum		−33	−73	−28		16							
		Middle occipital gyrus	18	−24	−97	−1	4.23				−24	−70	−25	3.91					−27	−64	−25									
		Premotor cortex	6	−15	14	47	4.22	100			−33	−70	−28	3.67																
			6	−12	8	62	4.09		Cerebellum		12	−39	−49	3.97	69															
			6	−21	11	56	3.92		Premotor cortex	6	54	−4	35	5.03	56															
		Fusiform	37	−51	−40	−10	4.09	46	Prefrontal cortex-SMA	8	42	5	35	3.74	11															
		Fusiform		−48	−52	−19	3.19																							
		Middle occipital gyrus	18	−3	−70	2	3.75	33																						
		Striate cortex	17	−18	−79	14	3.78	17																						

P2	Incorrectly named > baba	Superior parietal lobule	7	−21	−61	35	5.59	1273	Cerebellum		18	−25	−34	5.58	471	Trained > baba	Cerebellum		15	−73	−31	3.75	10	Premotor cortex	6	51	−4	35	4.25	38
		Primary motor	4	−3	−28	74	4.96		Cerebellum		9	−37	−49	4.75																
		Middle occipital gyrus	18	−27	−85	5	4.34		Angular gyrus	39	36	−58	44	3.91	76	Untrained > baba	No suprathreshold cluster							No suprathreshold cluster						
		Premotor cortex	6	−3	8	65	4.58	230	Superior parietal lobule	7	30	−61	35	3.6																
			6	−15	14	47	4.13		Primary motor	4	57	−1	32	4.37	39															
									Middle frontal gyrus	9	54	26	20	3.91	22															
									Inferior frontal gyrus	45	54	29	11	3.69																
									Anterior middle frontal gyrus	46	48	35	17	3.25																

BA: Brodmann area; baba: condition control.

**Table 4:** Lateralization indexes related to successful verb naming in the different conditions pre- and posttherapy for P1 and P2.

Lehéricy index	P1	P2
Spontaneous pretherapy	0.08	0.6
Spontaneous posttherapy	−1	−0.07
Incorrect pretherapy	−0.2	0.42
Incorrect—trained posttherapy	0.17	−0.58
Incorrect—untrained posttherapy	−0.73	N/A
